# Serum Cystatin C Levels as a Predictor of Severity and Mortality Among Patients With COVID-19 Infection

**DOI:** 10.7759/cureus.42003

**Published:** 2023-07-17

**Authors:** Kavya Prasad, Ashwin Kulkarni, Navikala K, Vanitha Gowda, Mohammed Aslam Shaikh

**Affiliations:** 1 Internal Medicine, Ramaiah Medical College, Bengaluru, IND; 2 Biochemistry, Ramaiah Medical College, Bengaluru, IND

**Keywords:** inflammation, covid-19 mortality, covid-19 severity, covid-19, serum cystatin c

## Abstract

Introduction: The pandemic caused by SARS Corona Virus-2 (COVID-19) has caused widespread mortality globally. The hallmark of the disease is the "cytokine storm," which is caused due to dysregulated immune system activation. Numerous inflammatory markers are used to predict the severity and mortality of the infection. Serum Cystatin C levels are associated with immune responses to exogenous and endogenous antigens. Our study was done to assess serum cystatin C as a marker of severity and mortality among patients admitted with COVID-19 infection.

Methodology: This cross-sectional study was conducted in a tertiary care center in South India. Sixty-nine patients with mild and severe COVID-19 infection admitted to the hospital were included in the study. Serum Cystatin C levels were estimated at admission. The levels were correlated with disease severity and mortality. Receiver operating characteristic curves (ROCs) was constructed for Cystatin C to predict severity and mortality. The computation of sensitivity, specificity, and positive and negative predictive values was done using optimal cut-off points. SPSS 18 was used for the statistical analysis. Version 18.0 of PASW Statistics for Windows. SPSS Inc., Chicago.

Results: Out of 69 patients, 28 (40.5%) had a mild illness, and 41 patients (59.4%) had severe COVID-19 illness. Mean serum Cystatin C levels measured at the time of admission among patients with mild illness was 1.83 (SD-1.53), and among patients with severe illness was 3.84 (SD- 2.59) (p<0.001). The area under receiver operating characteristic curves (ROC) for serum cystatin C for predicting COVID-19 severity and mortality was 0.904 and 0.768, respectively (p<0.001).

Conclusion: Patients with severe COVID-19 disease had considerably higher serum levels of Cystatin C than those with mild COVID-19 illness. Cystatin C levels can be useful for predicting mortality and severity among patients admitted with COVID-19 infection.

## Introduction

The pandemic caused by the highly infectious SARS-CoV-2 (COVID-19) has led to a global health crisis. COVID-19 is a multisystem disease that evokes a widespread inflammatory response known as a 'cytokine storm', which is responsible for the adverse outcome of illness. The inflammatory biomarkers can be important in early detection, progression, severity, and disease outcome [1]. Commonly used inflammatory biomarkers are C-reactive protein(CRP), Lactate dehydrogenase (LDH), serum ferritin, D-Dimer, etc. The serum levels of biomarkers are highly sensitive, not only detecting minor cases early but also predicting the severity of the disease, and can help clinicians to diagnose such cases earlier and subsequently initiate appropriate treatment measures [2].

Cystatin C is a low-molecular-weight protein that is extensively distributed in different organs. It is also used as a marker of glomerular filtration rate [3-5]. Numerous studies have shown a connection between cystatin C and the immunological responses to diverse endogenous and endogenous antigens. Evidence shows that different cytokines released during inflammation regulate the gene that codes for Cystatin C [5]. Some authors have suggested that cystatin C has a prognostic role in managing inflammatory diseases [5-6].

Similarly, the estimation of serum Cystatin C can be elevated in patients with COVID-19 infection and can reflect the inflammatory process associated with COVID-19 infection. Cystatin C is also known to modulate the release of numerous inflammatory cytokines like tumor necrosis factor-α, interleukin-12, interleukin-10, and nitric oxide (NO) [7-8]. These mediators are involved in the inflammation and subsequent multi-organ failure that can happen in severe COVID-19 infection [9-10]. The accuracy of these markers is variable, and studies have demonstrated that they do not predict the severity and mortality of COVID-19 infection [11].

This study assessed the serum Cystatin C levels as a predictor of severity and mortality among patients with COVID-19 infection.

## Materials and methods

This cross-sectional study was conducted at Ramaiah Medical College and Hospitals, Bengaluru, India. The study was conducted from January 2022 to March 2022. The study was carried out after obtaining approval from the institution's ethics committee (MSRMC/EC/SP-09/05-20220) and as per the Declaration of Helsinki 2013. The study included consecutive patients diagnosed with COVID-19 infection and admitted to the hospital during the study period. The study included a total of 69 adult patients aged more than 18 years. To confirm COVID-19 infection, a reverse transcriptase polymerase chain reaction (RT-PCR COVID-19) test was performed on a nasopharyngeal sample. Patients with known chronic kidney disease, hyperthyroidism, Autoimmune diseases, Current malignancies, Ongoing Chemotherapy, pregnancy, and patients who developed acute kidney injury during treatment were excluded. These cases were classified into mild, moderate, and severe COVID-19 infection per Indian Council of Medical Research guidelines (Table 1) [12].

**Table 1:** Classification of severity of COVID-19 infection.

Severity of COVID-19 illness	Description
Mild COVID-19 illness	Patients with fever and upper respiratory tract infection symptoms like cough, running nose, throat pain, and oxygen saturation of more than 94% at ambient air and respiratory rate less than 22 per minute.
Moderate COVID-19 illness	Patients with a respiratory rate of more than 24 breaths per minute or an oxygen saturation of less than 94% on ambient air were considered to have a moderate COVID-19 disease.
Severe COVID-19 illness	Patients with a respiratory rate greater than 30 per minute or an oxygen saturation below 90% on ambient air were considered to have severe disease.

For comparison, patients were divided into mild COVID-19 infection and severe COVID-19 infection (which also included moderate infection). Clinical details such as complete history and clinical examination were done. Blood samples were drawn from patients on admission before administering any medications. Around 5ml of blood was collected under strict aseptic precaution in a plain vacutainer with gel, mixed, and allowed to stand undisturbed for 5-8 mins. The samples collected were then stored at -20°C till Cystatin C analysis. Cystatin C level was estimated on Semi autoanalyzer (manufactured by Velocity Biosystems) by immunoturbidimetry in the Department of Biochemistry. Normal serum cystatin C levels using this method were 0.57-1.12 mg/dL. The data of complete blood count, C-reactive protein (CRP), D-Dimer, serum ferritin, and renal function tests were done, and the data was collected. Serum cystatin C level was measured at the time of admission, and all the patients were followed up till their stay in the hospital. Serum creatinine levels were measured and recorded. The Cockcroft gault formula estimated the glomerular filtration rate for all patients. The clinical course and outcome were followed up in the hospital.

Statistical analysis

Descriptive statistics of Cystatin C serum levels were analyzed and summarised in terms of mean with Standard Deviation. The severity and mortality of COVID-19 infection were summarised in terms of percentage. Receiver operating characteristic curves (ROCs) was constructed for Cystatin C to predict severity and mortality. The computation of sensitivity, specificity, and positive and negative predictive values was done using optimal cut-off points. A test with an area under the ROC curve of 0.5 predicts a result no better than by chance. An area under the ROC curve above 0.8 indicated a fairly accurate prediction. The p-value (Probability that the result is true) of 0.05 was regarded as statistically significant. Software developed by SPSS Inc. and released in 2009, SPSS 18, was used for the statistical analysis. Version 18.0 of PASW Statistics for Windows. SPSS Inc., Chicago.

Sample size with justification

Zinellu et al. (2021) observed that the sensitivity of predicting the death of patients with severe and critical cases of COVID-19 was 86.5, with a cut-off value of Cystatin C as 0.80 mg/dl [10]. In the present study, with a 95 confidence level and 9 margin of error, the study required a minimum of 55 subjects.

## Results

A total of 69 consecutive patients admitted during the study period were included. The number of males in the study was 42 (60.8%), and 27 (39.1%) were females. Twenty-six patients (38%) were between the age group of 71-80 years, and 17 (25%) were between 61 to 70 years. Table 2 shows the age distribution of the patients. Of 69 patients, 28 (40.5%) had a mild illness, and 41 (59.4%) had a severe infection. The description of the severity of COVID-19 illness in each age group has been summarized in Table 3. Fever was present in 24 (85.7%) patients with mild illness and 38(92.6%) patients with moderate to severe illness. Dyspnea was present in 37 (90.2%) patients with severe illness. The clinical presentation and comorbidities are summarized in Table 4. Mean serum Cystatin C levels among patients with mild illness were 1.83 (SD-1.53), and among patients with severe illness were 3.84 (SD- 2.59). This difference was found to be statistically significant. (P<0.001) The mean CRP, D-Dimer, and LDH among the patients with moderate and severe illness have been summarized in Table 5. Six patients (8.6%) of the patients included in the study died. These patients had severe COVID-19 illness at the time of admission.

**Table 2:** Distribution of patients as per age and gender

Age Group (years)	Male		Female	
	n	%	n	%
≤ 50	7	17%	6	22%
51-60	2	5%	3	11%
61-70	13	31%	4	15%
71-80	15	36%	11	41%
>80	5	12%	3	11%
Total	42	100	27	100

**Table 3:** Distribution of patients in each group according to age:

Age Group (years)	Mild COVID-19		Severe COVID-19	
	n	%	n	%
≤ 50	7	25%	6	15%
51-60	2	7%	3	7%
61-70	6	21%	11	27%
71-80	11	39%	15	37%
>80	2	7%	6	15%
Total	28	100	41	100

**Table 4:** Clinical presentation and comorbidities among the patients

Parameters	Mild COVID-19 Illness (n=28)	Severe COVID-19 illness (n=41)
Fever	24 (85.7%)	38 (92.6%)
Cough	24 (85.4%)	36 (87.8%)
Dyspnea	01 (3.5%)	37 (90.2%)
Loss of smell	15 (53.5%)	22 (53.6%)
Type-2 Diabetes mellitus	12 (42.8%)	25 (60.9%)
Hypertension	10 (35.7%)	26 (63.4%)
Ischemic heart disease	2 (7.1%)	14 (34.1%)

**Table 5:** Comparison of laboratory parameters between the patients with moderate and severe COVID-19 illness CRP- C-reactive protein; LDH- Lactate dehydrogenase; eGFR- estimated glomerular filtration rate

Parameter	Group	n	Mean	Std Dev	P-Value
CRP (mg/dL)	Mild	28	10.1	9.8	0.038
	Severe	41	16.5	10.1	
D Dimer (ng/ml)	Mild	26	2.1	2.4	0.344
	Severe	40	2.8	2.80	
IL-6	Mild	12	160.1	259.4	0.466
	Severe	22	276.5	509.5	
Ferritin (mg/dL)	Mild	28	325.7	286.7	0.656
	Severe	39	389.2	356.3	
LDH (mg/dL)	Mild	28	320.3	407.2	0.122
	Severe	41	348.5	172.6	
Serum Creatinine mg/dL	Mild	28	0.7	0.3	0.62
	Severe	41	0.9	0.5	
eGFR (ml/kg/1.73 m^2^)	Mild	28	95.4	3.5	0.45
	Severe	41	93.6	4.6	
Cystatin C Mg/dL	Mild	28	1.8	1.53	<0.001*
	Severe	41	3.8	2.39	

**Figure 1:**
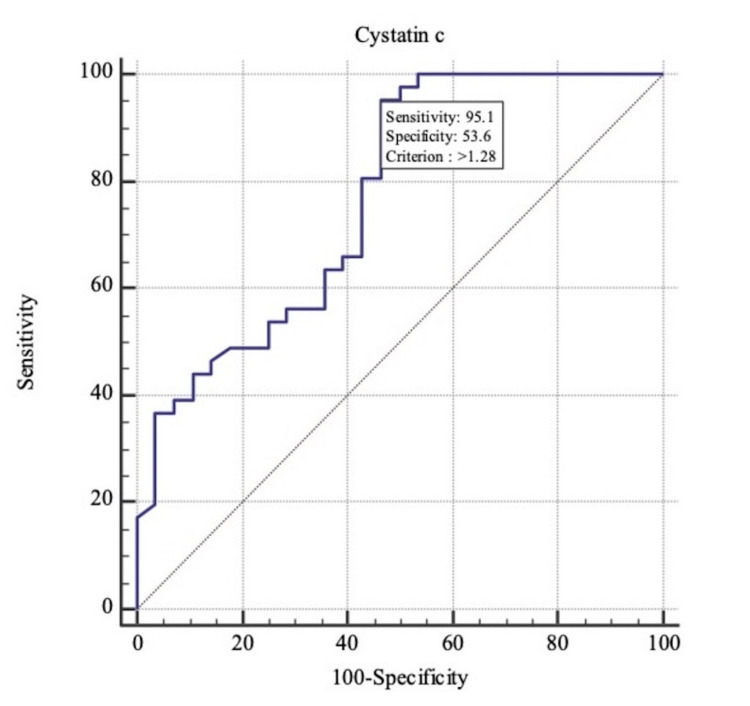
Receiver operating characteristic curve (ROC) for serum Cystatin C for predicting COVID-19 severity.

**Figure 2:**
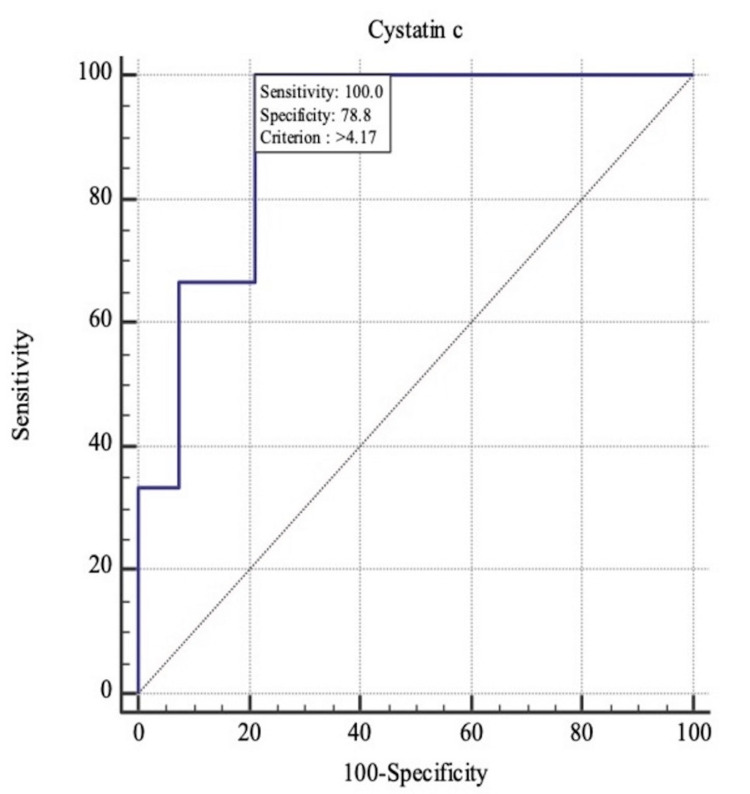
Receiver operating characteristic curve (ROC) for serum Cystatin C for predicting COVID-19 mortality.

The area under the ROC curve for serum cystatin C for predicting COVID-19 severity and mortality for 0.904 and 0.768, respectively (p<0.001) (Table 6, Figure 1-2). Serum Cystatin C level of more than 1.28mg/dL predicted COVID-19 severity with a sensitivity of 95.12% and specificity of 100%. Cystatin C levels of more than 4.17mg/dL predicted mortality with a sensitivity of 100% and specificity of 78.79%. (Table 7). Mean serum cystatin C among survivors was 1.48mg/dL, and among non-survivors was 4.28mg/dL (P<0.001).

**Table 6:** Area under the ROC curve for serum cystatin C predicting severity and mortality of COVID-19 infection. ROC- Receiver operating characteristic curve; AUC-Area under curve

Parameter	Severity of COVID-19	Mortality of COVID-19
Area under the ROC curve (AUC)	0.904	0.768
Standard Error	0.0668	0.0593
95% Confidence interval	0.809 to 0.962	0.651 to 0.861
P Value	<0.0001	<0.0001

**Table 7:** Optimal cut-off of serum cystatin C for predicting severity and mortality of COVID-19 infection with specificity, sensitivity, positive predictive value (+PV) and negative predictive value (-PV)

Parameter	Serum Cystatin C	Sensitivity	95% CI	Specificity	95% CI	+PV	-PV
Severity	>1.28	95.12	83.5 - 99.4	53.57	33.9 - 72.5	75.0	88.2
Mortality	>4.17	100.00	29.2 - 100.0	78.79	67.0 - 87.9	17.6	100.0

## Discussion

The current study demonstrated that serum levels of cystatin C done at the time of admission were statistically elevated among patients with severe COVID-19 illness compared to patients with mild COVID-19 illness. An interesting finding of the study was that even though other inflammatory markers like D-Dimer were higher among the patients with severe illness, the difference was not statistically significant in this study.

Cystatin C is a marker for acute renal injury and inflammation and has a role in releasing inflammatory markers [13]. However, the current study showed no statistical significance for serum creatinine levels between mild and severe illness. This shows that cystatin C levels can increase in severe inflammation.

In the current study, the mean serum cystatin C among survivors was 1.48mg/dL, and among non-survivors was 4.28mg/dL. This finding was similar to the meta-analysis by Zinellu A et al. involving thirteen studies in 2510 COVID-19 patients. The results showed that serum cystatin C levels were significantly elevated in patients with COVID-19 infection and patients who succumbed to the illness (standard mean deviation, SMD, 1.71, 95% CI 0.95 to 2.46, p < 0.001) [10].

The observations of the current study were similar to the study conducted by Li Y et al. in China, which demonstrated that serum cystatin C was an independent risk factor for mortality in patients admitted with COVID-19 infection (Odds ratio = 1.812, 95% confidence interval [CI]: 1.300-2.527, P < 0.001). The area under the ROC curve was 0.755 (95%CI: 1.300-2.527), the cut-off value was 0.80, the specificity was 0.562, and the sensitivity was 0.865 [14].

The current study's findings were similar to the study by Chen D et al., which demonstrated that the highest cystatin C level was significantly related to more severe inflammatory conditions, worse organ dysfunction, and worse outcomes among patients with COVID-19 ( values < 0.05). In the adjusted logistic regression analyses, the highest cystatin C level and ln-transformed cystatin C levels were independently associated with the risks of developing severe COVID-19 and all-cause mortality in overall patients or patients without chronic kidney disease (values < 0.05). The study demonstrated that patients with severe COVID-19 illness had elevated levels of serum Cystatin C [15].

As per the current study, the area under the ROC curve for serum cystatin C for predicting COVID-19 severity and mortality for 0.904 and 0.768, respectively (p<0.001). These findings were concordant with the study conducted by Lin L et al., where the area under the ROC curve was 0.708 (95% CI 0.594-0.822), the cut-off value was 1.245 (mg/L), and the sensitivity and specificity were 79.1% and 60.7%, respectively [16]. Similarly, as per the study by Kumar T et al., the area under the curve (AUC), optimal threshold, specificity, and sensitivity were 0.749, 0.888, 0.746, and 0.688, respectively. For mortality prediction, AUC, optimal threshold, specificity, and sensitivity for day 1 were 0.695, 1.04, 0.859, and 0.60, respectively [17].

Many studies have shown that Cystatin C can exert immunomodulatory effects by regulating the production and release of cysteine proteases [10]. Cystatin C is associated with various immune responses to internal and external antigens. This has led to many authors suggesting that Cystatin C can have prognostic use in predicting the outcomes of inflammatory diseases. Cystatin C can influence the release of many inflammatory cytokines like tumor necrosis factor-α, interleukin-12, interleukin-10, and nitric oxide. This leads to the production of highly reactive NO derivatives and can lead to nitrosative stress. These factors can cause irreversible cellular injury, apoptosis, and cellular dysfunction [13]. These processes play a vital role in the pathophysiology of the cytokine storm and multiple organ dysfunction, which can happen with severe COVID-19 illness. The study portrays the potential role of serum cystatin C in COVID-19 infection. This is the first such study conducted in India. Serum cystatin C levels can be helpful as a predictor of severity and mortality among patients admitted with COVID-19 infection. This study will pave the way for larger multicentric studies wherein Cystatin C can be evaluated as a potential biomarker that can help clinicians in the stratification of high-risk patients and early escalation of the treatment based on the disease outcome. The study's limitations were a relatively smaller sample size and the study being done in a single center.

## Conclusions

Patients with severe COVID-19 disease had considerably higher serum levels of cystatin C. Cystatin C levels can be useful as a predictor of mortality and severity among patients admitted with COVID-19 infection. This will help the treating physicians identify high-risk patients and promptly escalate the treatment.
